# Arbuscular mycorrhizal fungi spore density and species diversity varied within plants species and between dry Afromontane forests in northwestern Ethiopia

**DOI:** 10.1371/journal.pone.0329429

**Published:** 2026-09-03

**Authors:** Belay Berza Beyene, Workie Nibret Alamirew, Belayneh Asfaw Atnaf, Jemal Yimer Kebede, Marcela Claudia Pagano, Fassil Assefa Tuji

**Affiliations:** 1 Department of Biology, College of Natural and Computational Sciences, Debre Markos University, Debre Markos, Ethiopia; 2 Department of Microbial Sciences and Genetics, College of Natural and Computational Sciences Addis Ababa University, Addis Ababa, Ethiopia; 3 Department of Botany, Federal University of Minas Gerais, Belo Horizonte, Minas Gerais, Brazil; Friedrich Schiller University, GERMANY

## Abstract

Ethiopian Afromontane forests are rich in biodiversity and they a part of the eastern biodiversity hotspot. However, they are the most degraded ecosystems, continuously shrinking in size. Arbuscular mycorrhizal fungi (AMF) form a symbiotic association with land plants; thereby assist plants by absorbing nutrients and water beyond root depletion zones. Further they help plants in tolerating abiotic and biotic stresses. The objective of this study was to determine the AMF spore density and species diversity associated to the dominant plant species in Aradie and Zengena dry Afromontane forests. Transects were laid down in Aradie and Zengena dry Afromontane forests and 20 plots were selected from each forest. About 1 kg rhizosphere soil was collected from each plant species in December, January and February. AMF spore extraction was done by using wet sieving and decantation method. The mean AMF spore density was varied between 17.3 and 196 spores per 100 g dry soil in Aradie forest and it was between 84 and 234 spores per 100 g dry soil in Zengena forest. The highest AMF spore density was recorded from *Olea africana* (196 spores per 100 g dry soil) in Aradie forest and 234 spores per 100 g dry soil from *Cupressus lucitanica* in Zengena forest. Six AMF morphotypes belonging to three genera and 15 morphotypes belonging to four genera were recorded from Aradie and Zengena forests, respectively. Genus *Acaulospora* was dominantly recorded from 71.4% of plant species in Aradie forest and from 100% plants in Zengena forest. Plants *Acacia abyssinica* and *Cupressus lucitanica* harbored the highest AMF species. Hence, these plant species and AMF species associated to them can be used in the rehabilitation and restoration of dry Afromontane forests in Ethiopia.

## 1. Introduction

Ethiopia is one of the African countries endowed with a rich biological resources or biological diversities. The rich biological diversities enabled Ethiopia to be considered as one of the world’s biodiversity hotspots. It is also one of the 12 Vavilov centers of crop genetic diversity [1]. Ethiopian Afromontane forests are the biodiversity rich ecosystems, mostly occurring in the high mountain regions and are internationally recognized as the part of eastern Afromontane biodiversity hotspots [2]. Attributed to their biodiversity and carbon storage potentials, the dry Afromontane forests restoration is recognized to be among the global forest restoration priorities [3]. In agreement with the global forest restoration priorities and initiatives, Ethiopian dry Afromontane forest restoration is also a national ecosystem restoration priority [4]. However, the dry Afromontane forests are the most degraded duet human interference and severely affected forests. As a result of that they are continuously shrinking in their land cover. The continuous shrinking in the size of these forests is mainly because of deforestation for agricultural land expansion and grazing lands, leaving a highly fragmented forest patches around churches and the mountainous areas [5].

Forests are sources of germplasm for biodiversity conservation and are examples of conserving exceptionally high biodiversity [6]. Hence, restoration of the degraded forest ecosystem can be achieved, if both the above ground and below ground biodiversity is effectively conserved, including the microorganisms. Therefore, a close linkage of the above and below ground soil microorganisms with that of above ground plants and below micro and macro biota help to build a healthy ecosystem in general and forest ecosystem in particular [6,7].The productivity of plant communities depend on the symbiotic effectiveness of the colonizing fungi and the diversity of fungal assemblage [5]. In turn, the distribution and abundance of fungi are strongly affected by habitat conditions, diversity and abundance of host plants [7].

AMF are one of the most important groups of below-ground biota which form a symbiotic association with more than 80% of the land plants [8]. In the symbiosis, the plant supplies the fungus with photosynthetic products for growth and reproduction and the fungus in turn provides the plant and soil with several benefits such as expansion of the nutrient absorption areas beyond the root depletion zone and thereby increase the uptake of water and nutrients (phosphorus in particular) [9,10]. AMF also help plants to improve tolerance to various forms of biotic and abiotic stresses [11,12]. In addition, AMF accumulate carbon [13] and contribute to the increase of microbial biomass in the soil, favoring carbon sequestration process. They further contribute to the formation and stability of soil aggregates by the production of glomalin [14].

Factors affecting AMF species diversity and distribution include plant species, soil type and its physical and chemical properties, climate (at global and regional level), topography at local scale and disturbance [6]. AMF contribution is more important in degraded and marginal soils in arid and semi-arid regions, where water and nutrients are limiting the plant growth [15].In this context, inoculation of tree seedlings with AMF species has a great potential for the rehabilitation and restoration of degraded lands. AMF inoculation further improves seedling establishment and success rate due to their role in nutrient and water absorption and translocation. Moreover, AMF help seedlings share growth limiting plant nutrients via common mycorrhizal networks [15].

Forests serve as a source of the AMF propagule spore banks, abundance, and diversity and infectivity information. They can also serve as control laboratories for studying effects of soil degradation and disturbances on microbial community structure and composition. Moreover, forests can serve as inoculants sources for ecologically, agriculturally and industrially important microorganisms.

In this context, there is dearth of scientific information on the AMF species diversity and population density associated with plant species in the Ethiopian Afromontane forests. Therefore, understanding and documenting the AMF species diversity and population density in such forest patches can be used as inoculants for enhancing restoration and rehabilitation of degraded and disturbed forests. To the best of our knowledge, there is no scientific information regarding the AMF spore density and species diversity in dry Afromontane forest patches of Dega Damot (Aradie) and Zengena in north western Ethiopia. The hypothesis of this study is that AMF spore density and species diversity are varied among associated host plant species and between forests. Therefore, the objective of this study was to determine the AMF spore density, species diversity, abundance and community composition associated with the plant species in Aradie and Zengena dry Afromontane forests in north western Ethiopia.

## 2. Materials and methods

### 2.1. Description of the study locations

**Fig 1:**
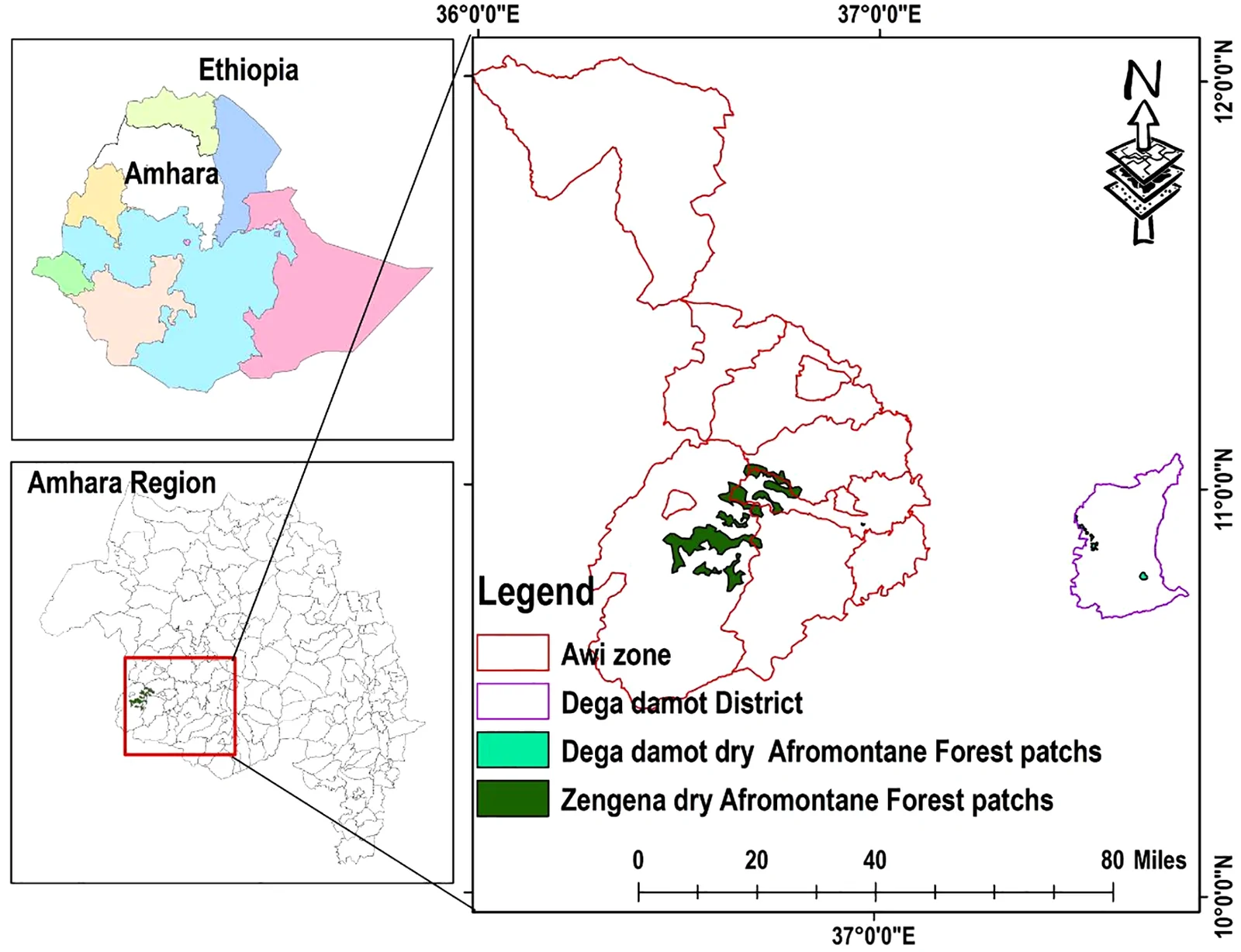
Map of the study areas.

This study was conducted in two fragmented remnant dry evergreen Afromontane natural forest patches (Aradie forest patch) in Dega Damot (West Gojam zone) and Zengena forest patch in the Banja district (Awi zone) in the Amhara National Regional State northwest Ethiopia (Fig 1).

Dega Damot (Aradie) dry Afromontane natural forest patch is located at 1^0°^ 40’ and 11° 5’ N latitude and 37° 29’ E and 37° 46’ E. The forest is 400 km far from Addis Ababa in the northwest direction. The district has an altitude ranging from 2300 to 3400 m.a.s.l. About 70% of the study area is characterized as Dega with the annual rainfall between 1,116.6−2,784.64 mm, and the mean annual rainfall is about 1774.7 mm. The average annual temperature of the district was between 10–16 °C [16]. In Dega Damot district, the dry season occurs between November and March, while the wet season occurs between April and October [17].

The Zengena forest patch is located at 1^0°^ 54’ N latitude and 36° 58’ E. The Zengena forest surrounds the Zengena lake, a crater lake with an areas of 25 ha and a depth of 160 m [18]. The Zengena forest has an altitude between 2,470–2585 m.a.s.l with the area of 68 ha. It has a tropical highland climate, which is dominated by a dry season. Zengena forest (Awi zone) is classified as an intermediate evergreen Afromontane or transition between dry and moist Afromontane forests [19]. The main rain season in Zengena starts in mid June and extends to mid October with the highest rainfall occurring between July and August. The mean annual rainfall ranges between 1,300 and 1,800 mm and the mean annual temperature ranges between 16 and 20 °C. The Aradie and Zengena forests are situated in different locations, with a distance of 127 kilometers separating them.

### 2.2. Plant species/Vegetation sampling

A preliminary survey was made at the both forests and transects were laid down into four directions starting from the centre of the each forest. Based on the distribution of plant species, we selected 20 plots from each forest (10 m x 10 m) across each transect at a distance of 100 m in each forest and plant species within the plots were counted [20]. The dominant plant species were sampled from each forest following the transect method [21]. Seven (at Aradie) and (five at Zengena) dominant plant species were considered from each plot. A triplicate of samples was collected and analyzed for each plant species from both forests. The local names of the plant species were recorded and the voucher specimen were pressed and then transported to the herbarium at Debre Markos University for the plant species scientific name identification. The list of dominant plant species included in the study is presented in the Table 1.

**Table 1:** The plant species sampled from Aradie and Zengena Afromontane forest patches in the northwestern Ethiopia.

Aradie Forest patch				Zengena forest patch			
Scientific name	Local name	Family name	Habit	Scientific name	Local name	Family name	Habit
*Osyris quadripartita*	Keret	Santalaceae	Shrub	*Casuarina cunninghamiana*	Arzelibanose	*Casuarinaceae*	Tree
*Rose abyssinica*	Kega	Rosaceae	Shrub	*Acacia abyssinica*	Girar	*Fabaceae*	Tree
*Apodytes dimidiate*	Dong(Cheleglegga)	Icacinaceae	Tree	*Cupressus lusitanica*	_	*Cupressaceae*	Tree
*Bersama abyssinica*	Azamera	Melianthaceae	Shrub	*Dovyalls caffra*	Askwar	*Flacourtiaceae*	Tree
*Erica aborea*	Aseta	Ericaceae	Shrub	*Prunus africana*	Tikurinchet	*Rosaceae*	Tree
*Olea europea*	Woyra	Oleaceae	Tree	*Vangueria volkensii*	Dingaiseber	*Rubiaceae*	Tree/shrub
*Eucalyptus globules*	Bahirzaf	Myrtaceae	Tree	–	–	–	–

### 2.3. Rhizosphere soil sampling

Seven dominant plant species (from Aradie) and five dominant plant species (from Zengena) were selected from each plot in both forests and grasses surrounding the plants were cleared. About 1 kg soil samples were collected from the rhizosphere of plants at each plot in both forests by digging up to 0–20 cm depth. The soil sampling was carried out during the dry season starting in the December 2022 and ended in February 2023. The soils of Dega Damot district are classified and belong to the chromic luvisols [22]. Similarly, the soils in Banja district are classified and belong to the andisols [23]. Soil samples from the same plant species in the same plot in the same forest were pooled into one composite sample. A triplicate of composite samples was considered for AMF spore extraction. The soil samples were carried to the Department of Biology, Mycology and Microbiology laboratories using sterile polythene bags. The soil samples were subdivided into two and 500 g was used for the soil physiochemical parameters analysis and the other 500 g was used for the AMF spore extraction.

### 2.4. Soil physicochemical analysis

The physicochemical analysis of the rhizosphere soil samples was conducted following standard methods and procedures at soil, water and plant sample analysis laboratory of Engineering Cooperation of Oromo (ECO), Addis Ababa, Ethiopia. About 500 g air-dried soil samples were grounded and sieved by using 1 mm sieve. The soil pH was determined on 1:2.5 (soil: water) suspension method [24]. The soil organic carbon was determined according to the methods and procedures described in the Walkley and Black method [25]. The total nitrogen (TN) was determined according to the Kjeldahl method [26]. The available phosphorus was determined according to the Olson method [27].

### 2.5. AMF spore extraction

The AMF spores from the rhizosphere soil samples were extracted using wet sieving and decantation method according to Gedermann and Nicolson [28]. A 100 g of air-dried soil was suspended in 500 mL of tap water and left for about 1–2 h in triplicates. The coarse soil particles were gently crushed by hand and left for 30 min to settle down. In addition, the soil samples were soaked in 6.3 mMolar sodium hexa-metaphosphate to disperse the clay fractions. The suspension was passed and washed through a series of four sieves with different sieve pore sizes arranged from top to bottom (500, 212, 90, and 45 µm), respectively. The washing process was repeated several times until the suspension become clean. The residues from 500 µm sieve were checked for the presence of mega spores, sporo-carps and spore clusters and were discarded in the absence and included into the smaller sieves (45 µm), if the mega spore(s),spore cluster(s) and/or sporo-carp(s) is/are available. The residues from 212 and 90 µm sieves were washed into a 45 µm sieve. Finally the residues in 45 µm sieves were transferred to 15 ml falcon tubes and centrifuged at 2000 rpm for 5 min. The supernatant was poured off, and pellets were suspended in 50% sucrose solution (w/v) and centrifuged again at 2000 rpm for 3 min. The supernatant was collected into the 45 µm sieve and the sucrose was washed-removed by using tap water. Finally spores, spore clusters and sporo-carps were collected and transferred into 90 mm diameter gridded plastic plates

### 2.6. Enumeration of AMF spores

Enumeration of AMF spores from each subsample was carried out under stereomicroscope at x40 magnification according to the methods and procedures described in the International Culture Collection of Vesicular-Arbuscular Mycorrhizal Fungi (INVAM), (http://invam.wu.edu/). In brief, for the enumeration and calculation of AMF spores, we used the diameter (mm) of the ocular lens and the base of the plates. Total number of fields in the plates was calculated as: Total number of fields = the area of the dish/the area of the lens. The total number of spores was counted from 40 randomly selected fields. The average number of spores per field was multiplied by total number fields to calculate total number of spores.

### 2.7. Identification of arbuscular mycorrhizal fungi spores

Healthy and intact spores were picked up under the dissecting microscope with a micropipette and mounted on a microscope slides permanently by using Polyvinyl-Lactic acid-glycerol (PVLG). They were also crushed and dried for two to three weeks at room temperature. Crushed spores were examined under a compound microscope (Olympus-BX51, Japan) at a magnification of x400 and pictures were captured. AMF spores were identified to species and/or genus level at Applied Microbiology Laboratory, Addis Ababa University, Addis Ababa, Ethiopia and Federal University of Minas Gerais, Brazil. Spores were identified and classified to genus/species level based on spore color, surface ornamentation, wall structure and subtending hyphae based on AMF species description and identification manual [29] and reference to the descriptions provided by INVAM (http://invam.wu.edu/) and (http://www.zor.zut.edu.pl/Glomeromycota/).

### 2.8. Data analysis

The assumptions of normality and homogeneity of variance were confirmed via the Shapiro-Wilk test (p > 0.05) and Levene’s test (p > 0.05), respectively. The number of spores per 100 g dry soil associated with each plant species in each forest was evaluated by using a one-way ANOVA followed by Tukey’s HSD post-hoc test for multiple comparisons (p < 0.05) by using SAS version 9.4. All data are presented as Mean ± SD. The AMF species or genus richness (SR), isolation frequency (IF), relative abundance (RA) and importance value (IV) were used to evaluate the structure of AM fungal communities in the rhizosphere of different plant species in the Aradie and Zengena dry Afromontane forest patches. These indices were computed as follows: Species richness (SR) is measured as the total number of identified AMF species per soil sample. Isolation frequency (IF) reflects the distribution status of an AM fungal species and calculated as (the number of samples in which a particular AMF species was observed/the total number of samples) × 100. The AMF species were classified into the following groups according to Chen et al. [30]: dominant (IF > 50%), most common (IF; 31%−50%), common (IF; 10%−30%), and rare (IF < 10%). Relative abundance reveals how strong or weak sporulation ability of different AM fungi. It is calculated as (the number of spores of a particular genus)/total number of identified spores) × 100.

## 3. Results

### 3.1. The plant species distribution and soil physicochemical characteristics in the forests

The forests are geographically separate and the distance between them is more than 127 Km. Even though Aradie and Zengena forests are Afromontane forest classes, we did not record the same dominant plant species from these forests. The dominant plant community in Aradie forest is completely different from the plant community in Zengena forest (Table 1). Plant rhizosphere soil was collected from these two dry Afromontane forest patches (Aradie and Zengena) and analyzed for selected soil physicochemical parameters. Soils at Aradie forest exhibited pH of 6.08, which is considered as slightly acidic, whereas those soils in Zengena forest showed pH value of 5.25, which is moderately acidic. The soil at Aradie forest was characterized by 5.93% organic carbon, 10.20% organic matter, 0.54% total nitrogen and 7.50 ppm available phosphorous. However, soils in the Zengena forest consisted of 3.57% organic carbon, 6.14% organic matter, 0.36% total nitrogen, and 12.58 ppm available phosphorous (Table 2).

**Table 2:** Soil physicochemical characteristics of the Aradie and Zengena forest patches.

Soil parameter (unit)	Aradie forest	Zengena forest
pH (1:2.5;soil:water)	6.08	5.25
Organic carbon (%)	5.93	3.57
Organic matter (%)	10.20	6.14
Total nitrogen (%)	0.54	0.36
Available P (ppm)	7.50	12.58

### 3.2. AMF spore density associated to the Afromontane forest patches

**Fig 2:**
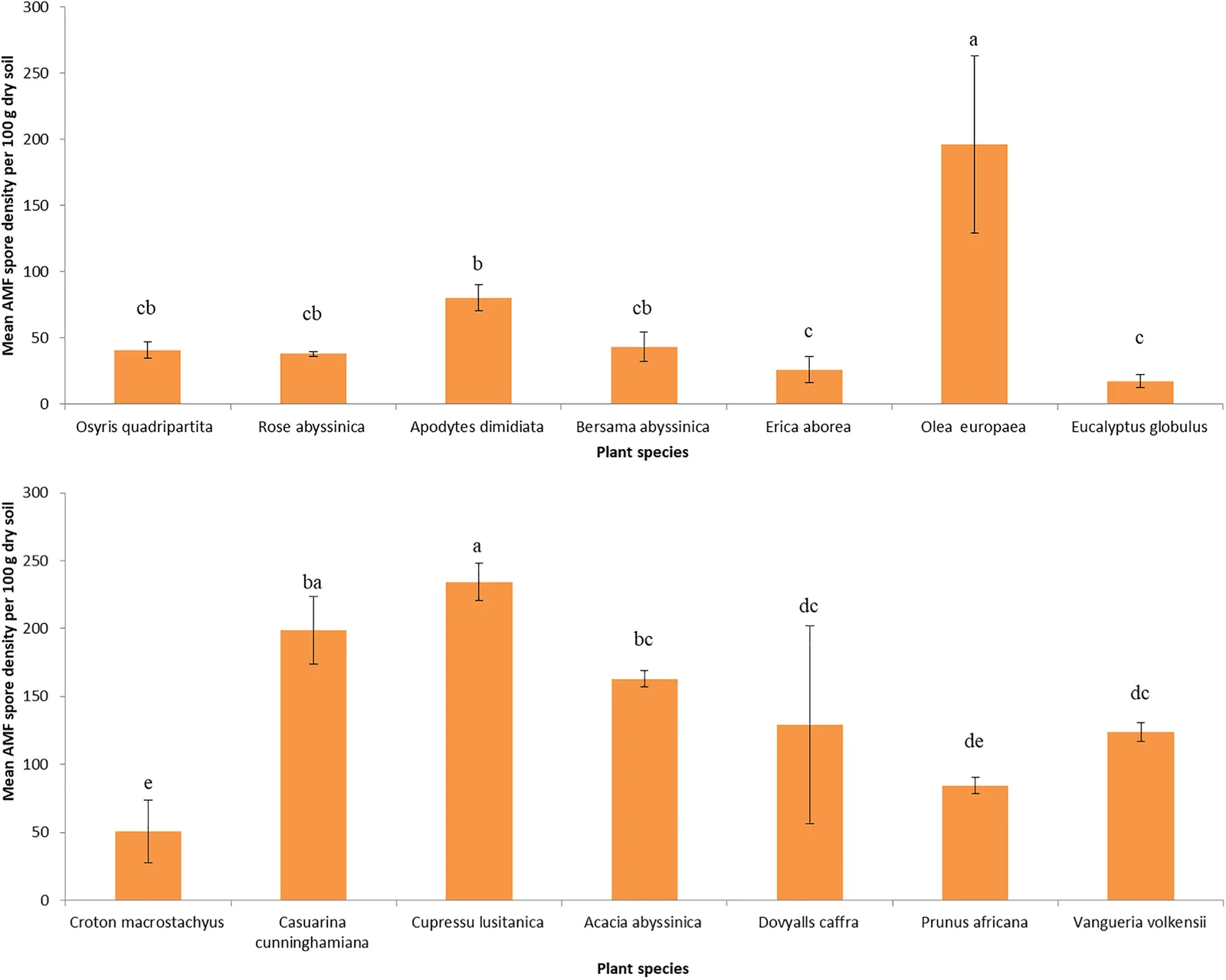
(A) AMF spore density in the rhizosphere of plant species Aradie forest. (B) Mean AMF spore density in the rhizosphere of plant species in Zengena forest.

In this study different plant species exhibited significantly (p < 0.05) different mean AMF spore density per 100 g dry soil in both Afromontane forests patches. The mean AMF spore density recorded from the rhizosphere of plant species in Aradie forest was varied between 17.3 spores per 100 g of dry soil in the rhizosphere of *Eucalyptus globules* plant and 196 spores per 100 g dry soil in the rhizosphere of *Olea europea* (Fig 2A).

The highest mean AMF spore density per 100 g dry soil (196) was recorded from the rhizosphere of *Olea europea* followed by 80.2 mean AMF spores per 100 g dry soil from the rhizosphere of *Apodytes dimidiate*. However, the lowest mean AMF spore density (17.3 spores per 100 g dry soil) was recorded from the rhizosphere of *Eucalyptus globules*. Similarly, the mean AMF spore density recorded from the rhizosphere plant species in the Zengena forest was significantly (p < 0.05) varied between 84–234 spores per 100 g dry soil. The highest mean spore density was recorded from the rhizosphere of *Cupressus lucitanica* (234 spores per 100 g dry soil), followed by *Casuarina cunninghmiana* (198.8 spores per 100 g dry soil), whereas the lowest mean spore density was recorded from *Prunus africana* (84 spores per 100 g dry soil) rhizosphere (Fig 2B).

**Fig 3:**
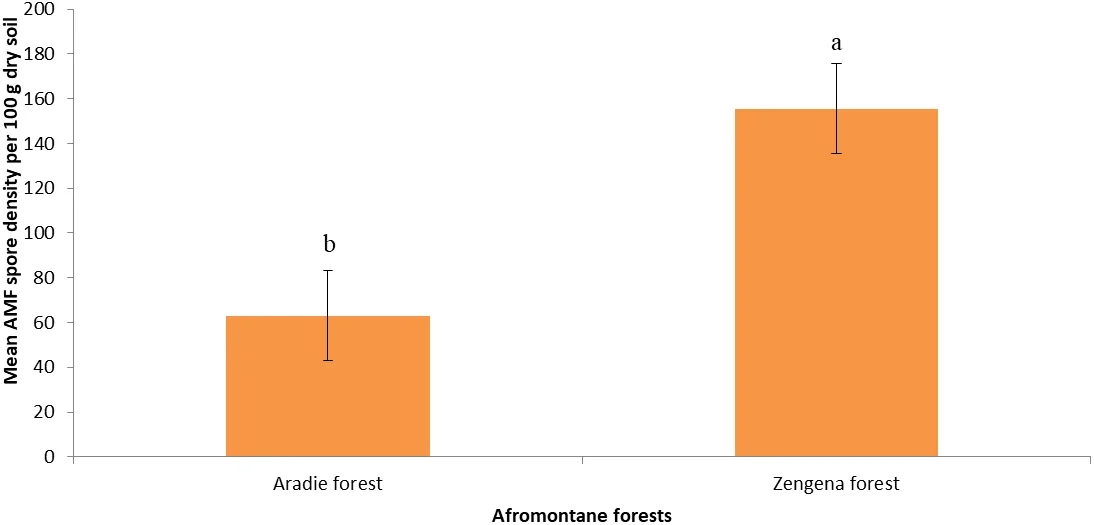
Mean AMF spore density in the Aradie and Zengena Afromontane forests.

The mean AMF spore density at forest level was significantly (p < 0.05) varied. The mean AMF spore density from Zengena forest was significantly (p < 0.05) higher (155.6 spores per 100 g dry soil) compared to the mean AMF spore density in Aradie forest (63 spores per 100 g dry soil) (Fig 3).

### 3.3. AMF genus and species composition associated to plant species in both forests

In Aradie forest, six (6) AMF morphotypes belonging to 3 genera were identified. Among these six morphotypes, four morphotypes (66.6%), one morphotype (16.6%) and another one morphotype (16.6%) were classified to *Acaulospora, Pacispora* and *Scutellospora,* respectively (Table 3).

**Table 3:** Isolation frequency (IF), relative abundance (RA) and importance value (IV) of AMF species.

Aradie forest (Dega Damot district)					Zengena forest (Banjaw district)				
Genus name	IF (%)	RA (%)	IV (%)	Status	Genus name	IF (%)	RA (%)	IV (%)	Status
*Acaulospora*	71.4	71.4	71.4	Dominant	*Acaulospora*	100	77.8	88.9	Dominant
*Pacispora*	14.3	14.3	14.3	Common	*Glomus*	16.7	11	13.9	Common
*Scutellospora*	14.3	14.3	14.3	Common	*Pacispora*	16.7	5.6	11.5	Common
–	–	–	–	–	*Racocetra*	16.7	5.6	11.5	Common

Among these morphotypes, about 66.6% were identified to the species level, whereas 33.3% were identified to the genus level (Table 4).

**Table 4:** Isolation frequency, relative abundance and importance value of AMF species from Aradie forest.

AMF species	IF (%)	RA (%)	IV (%)	Status
*Acaulospora fovea*	28.6	28.6	28.6	Common
*Acaulospora hemi*	14.3	14.3	14.3	Common
*Acaulospora* sp.1	14.3	14.3	14.3	Common
*Acaulospora* sp.2	14.3	14.3	14.3	Common
*Pacispora dominikii*	14.3	14.3	14.3	Common
*Scutellospora biornata*	14.3	14.3	14.3	Common

In Aradie forest, *Acaulospora* was isolated from the rhizosphere of 71.4% of the plant species, whereas genus *Pacispora* and genus *Scutellospora* were least recovered genera. Therefore, Genus *Acaulospora* was the dominant genus in the Aradie forest (Table 3).

Similarly, in Zengena forest, we have recorded 15 AMF morphotypes belonging to four genera. In this forest, genus *Acaulospora* was recovered from all the rhizosphere soils (100%) and hence the dominant genus in both forests, whereas the other genera were common in the forest (Table 3). From Zengena forest, we have identified (11) eleven morphotypes (73.3%) to the species level and four (4) morphotypes (26.7%) to the genus level. These 15 morphotypes recovered from Zengena forest include 11 morphotypes belonging to genus *Acaulospora*; two morphotypes belong to genus *Glomus*, one morphotype to belong genus *Pacispora* and one morphotype belong to genus *Racocetra* (Table 5).

**Table 5:** Isolation frequency, relative abundance and importance value of AMF species from Zengena forest.

AMF species	IF (%)	RA (%)	IV (%)	Status
*Acaulospora lacunose*	16.7	11	13.9	Common
*Acaulospora mellea*	33.3	11	22.2	Common
*Acaulospora myriocarpa*	16.7	5.6	11.2	Common
*Acaulospora scrobiculata*	33.3	11	22.2	Common
*Acaulospora* sp.1	16.7	5.6	11.2	Common
*Acaulospora* sp.2	16.7	5.6	11.2	Common
*Acaulospora* sp.3	16.7	5.6	11.2	Common
*Acaulospora* sp.4	16.7	5.6	11.2	Common
*Acaulospora* sp.5	16.7	5.6	11.2	Common
*Acaulospora* sp.6	16.7	5.6	11.2	Common
*Acaulospora* sp.7	16.7	5.6	11.2	Common
*Glomus* sp.1	16.7	5.6	11.2	Common
*Glomus* sp.2	16.7	5.6	11.2	Common
*Pacispora* sp.1	16.7	5.6	11.2	Common
*Racocetra castanea*	16.7	5.6	11.2	Common

### 3.4. AMF species richness associated to plant species in Aradie and Zengena forests

In Aradie forest, both AMF genus richness and species richness were the same (one genus or species per plant) in different plant species. That is, all the different plant species included in this study in the Aradie forest have equal number of AMF Species richness (SR) (Table 6).

**Table 6:** AMF genus richness, number of genus and species richness (SR) per plant species in the Aradie and Zengena forests.

Aradie forest patch					Zengena forest patch				
Plant species	Genus richness	Genus name	Number of each genus	Species richness	Plant species	Genus richness	Genus name	Number of each genus	Species richness
*Apodnoytes dimidiata*	1	*Acaulospora*	1	1	*Casuarina cunninghamiana*	1	*Acaulospora*	1	1
*Bersama abyssinica*	1	*Acaulospora*	1	1	*Acacia abyssinica*	2	*Acaulospora*	4	6
							*Glomus*	2	
*Erica aborea*	1	*Pacispora*	1	1	*Cupressus lusitanica*	2	*Acaulospora*	3	4
							*Racocetra*	1	
*Eucalyptus globules*	1	*Acaulospora*	1	1	*Dovyalls caffra*	2	*Acaulospora*	1	2
							*Pacispora*	1	
*Olea europea*	1	*Acaulospora*	1	1	*Prunus africana*	1	*Acaulospora*	1	1
*Osyris quadripartita*	1	*Scutellospora*	1	1	*Vangueria volkensii*	1	*Acaulospora*	1	1
*Rose abyssinica*	1	*Acaulospora*	1	1	–	–	–	–	–

However, in Zengena forest, we have observed variations in species richness among plant species. In this context, AMF genus richness among plant species was varied from 1 to 2. The highest AMF genus richness was recorded and associated to *Acacia abyssinica*, *Cupressus lucitanica* and *Dovyalls caffra* were equal in number of genus richness (2). The lowest number of genus richness was recorded from *Casuarina cunninghamiana*, *Prunus africana* and *Vangueria volkensii* (1) (Table 6). Genus *Acaulospora* was associated to five plant species out of six plant species, which was about 83.3% of plant species. Regarding AMF species richness in Zengena forest, the highest AMF species richness was recorded from *Acacia abyssinica* (40%), followed by *Cupressus lusitanica* (26.6%) (Table 6).

## 4. Discussion

### 4.1. Distribution of dominant plant species in Aradie and Zengena forests

In this study, we have recorded completely different dominant plant species in both Aradie and Zengena Afromontane forest patches. That is, none of the dominant plant species were common to both Aradie and Zengena forests. This may be due to the fact that plant community distribution is highly affected by edaphic factors and topography at local scale and these factors play critical role in controlling plant community formation [17,31]. In addition, the difference in the plant community in the forests could be attributed to the fact that the Banja district is a transition or intermediate vegetation between dry Afromontane and moist Afromontane vegetation in North West Ethiopia [19]. Therefore, plant community formation is influenced by soil type (chromic luvisols at Aradie forest and andisols at Zengena forest), soil physical and chemical properties and topographic features at local scale [32,33]. The soil physical and chemical properties were varied in the forests (Table 2) and topographically, the altitudinal range of Aradie forest is between 2300–3400 m.a.s.l (difference;1,100 m.a.s.l), whereas Zengena forest ranges between 2400–2585 m.a.s.l. (difference; 285 m.a.s.l). Therefore, the differences in the dominant plant community composition in Aradie and Zengena forests could be attributed to these edaphic and topographic disparities.

### 4.2. Soil physicochemical characteristics

Soil nutrients are not only crucial for plant growth and productivity, but also important for the growth, reproduction and proper functioning of soil micro and macro biota in general. Even though these forests are dry evergreen Afromontane forests, they have exhibited varied soil physicochemical characteristics. For instance, soils in Aradie dry Afromontane forest were slightly acidic (pH; 6.08); however, those soils in Zengena forest were moderately acidic (pH; 5.25). The difference in pH of the two forests could be associated with soil formation, base saturation, climate; leaching and agricultural activities surround the forests. We have recorded a higher organic carbon and total nitrogen in the Aradie forest compared to the Zengena forest. This difference could be attributed to the difference in the soil biological activities and health in both forests. However, a higher available phosphorous was recorded in the Zengena forest (Table 2) compared to the Aradie forest. Our results exhibited slightly lower or moderately acidic pH compared to Birhane et al [7,34].from dry Afromontane forests in north Ethiopia. In another study, Birhane et al [6] have recorded a comparable soil pH from fragmented church natural forests in north Ethiopia, which is comparable to our results from Zengena dry Afromontane forest (Table 2). Similarly, the soil organic matter content in the present study was comparable to those recorded from fragmented church natural forests and dry Afromontane forests in north Ethiopia, respectively [6,34]. The soil available P in the present study locations was twice lower compared to those reported by Birhane et al. [6] from the fragmented church natural forests in north Ethiopia. The differences in soil physicochemical parameters in the present study and those from north Ethiopia may be attributed to difference in soil type, plant community compositions, soil microbial activity and topography.

In addition, the soil pH in both forests was much lower as compared to soil pH in Munessa Afromontane natural forest (7.45) in Oromia National Regional State in Shashemane district [35]. Similarly, Yimer *et al* [35] have reported 11.5% soil organic carbon, 19.8% soil organic matter, and 1.25% total nitrogen, which is much higher as compared to our results.

### 4.3. AMF spore density associated to the plant species in Aradie and Zengena forests

In the present study, we had recorded significantly (p < 0.05) different mean AMF spore density per 100 g dry soil associated with plant species in Aradie and Zengena forests. Much lower mean AMF spore density (63.1 spores per 100 g dry soil) was recorded in the Aradie forest compared to the higher mean spore density in Zengena forest (155.6 spores per 100 g dry soil) (Fig 3). The disparity in the mean AMF spore density per 100 g dry soil could be attributed to the difference in soil type (chromic luvisols in Aradie and Andisols in Zengena), soil physicochemical properties, host plant species, local climate and topography features [36]. Moreover, Zengena dry Afromontane forest is a transition forest between dry and moist Afromontane forests in Ethiopia [19]. Results in present study are in line with the spore densities recorded by Birhane et al. [6] from the fragmented church natural forest remnants in northern Ethiopia. However, the mean AMF spore density in the present study was higher as compared to those reported from land use types in different parts of Ethiopia: Muleta et al. [37] and [38] from coffee shade trees of Bonga natural forest and from smallholder agro-forestry and mono-cultural coffee systems in southwestern Ethiopia, respectively and Belay et al. [39] from native forests and agro ecosystems in southern Ethiopia. But, the mean AMF spore density in Aradie forest (in this study) was lower than mean AMF spores reported in different parts of Ethiopia: Berza et al. [40] from the rhizosphere of *Erythrina* species in Ethiopia and Dobo et al. [41] from the rhizosphere of different plant species in Sidama region, southern Ethiopia. Similarly, Birhane etal*,.*[7] have reported much higher AMF spore density per 100 g of soil in *Acacia abyssinica* rhizosphere (1127) in fragmented church natural forest remnants in northern Ethiopia. The differences in mean AMF spore density between forests in the present study as well as among other studies could be attributed to soil available phosphorous, soil pH, soil moisture and elevation [36]. In general variations in mean AMF spore density could also be attributed to the factors such as sampling season, soil chemical properties, Vegetation type, climatic condition and disturbances [42]. Birhane *et al*. [43] also suggested protecting fields from disturbances had positive effect for improving spore abundances.

### 4.4. AMF community composition of Aradie and Zengena forests

AMF are symbiotic fungi that colonize plant roots. They enhance nutrient uptake and their diversity influences forest productivity, resilience and plant community structure [40]. AMF species diversity was varied between Aradie and Zengena dry Afromontane forests. In support to the higher mean AMF spore density recorded in Zengena forest, we have also recorded higher AMF morpotypes (15) in Zengena forest compared to the Aradie forest (6). The higher AMF species diversity in Zengena forest as compared to Aradie forest could be attributed to the soil types and their chemical properties, climatic conditions, plant community composition, plant productivity and carbon allocation, topographic features and disturbances [39]. The AMF species diversity in present study is lower compared to Belay et al. [39] and [44] who have reported 37 AMF morphotypes in southern Ethiopia natural forest and 41 AMF species from Acacia trees in different land use system in Ethiopia, respectively. Similarly, Berza et al*.* [40] have also reported 11 genera and 33 AMF species associated to *Erythrina* species in different land use types of Ethiopia.

In this study, *Acaulospora* was found to be dominant in both dry Afromontane forests. The dominance of genus *Acaulospora* in Ethiopian soils has been previously reported by several scholars. For instance, Muleta et al. [37,38] have reported *Acaulospora*’s dominance in Bonga and Yayu natural forests in Southwestern Ethiopia. Similarly, Birhane et al., [7] and Dobo et al. [41] have reported *Acaulospora*’s dominance from Afromontane forest patches in northern Ethiopia and Sidama agro-forestry system in southern Ethiopia, respectively. Among AMF species, those species belonging to *Acaulospora* were widely distributed to worldwide across a variety of natural environments which could be attributed to their generalist nature, their great adaptability and high infectivity rate of their propagates [45]

Regarding AMF genus and species diversity, our results are comparable to kaonongbua [46] who have also identified 13 AMF species belonging to 7 genera from oil palm plantations in Thailand. However, some authors have reported a higher AMF morphotypes from similar studies. For example, Shi et al. [47] have reported higher (66 AMF species) belonging to 8 genera from medicinal plants in south East Asia, Belay et al. [39] and [44] have also reported 37 AMF morphotypes from natural forests in southern Ethiopia and 41 AMF species from *Acacia* trees in different land use system in Ethiopia, respectively. Similarly, Berza et al. [40] have also reported 33 AMF species belonging to 11 genera associated to *Erythrina* species in different land use types of Ethiopia.

Morphological identification (mainly based on spore traits) has been foundational but it comes with several important limitations. The drawbacks could include limited diagnostic features (many species share very similar morphological traits), phenotypic plasticity (morphological traits can vary depending on host plants and environmental conditions), there are cryptic species (morphologically identical spores may represent genetically distinct) and incomplete life stage observation (some species sporulate rarely leading to underrepresentation of diversity). To overcome these limitations, now day, molecular approaches are increasingly used as they provide higher resolution and accuracy, detection of non-sporulating taxa, reduce subjectivity and assist discovery of cryptic diversity.

The wide distribution of *Acaulospora* in present study makes them the most potential candidate AMF genera for mass multiplication and to use them as inoculants in plantation forest development programs and rehabilitation and restoration of degraded Afromontane forests in Ethiopia. Such potential AMF genera can be inoculated to the seedlings of trees in re-vegetation of degraded lands, which could improve establishment of seedlings in infertile and marginal soils.

## 5. Conclusion

This study underscores the critical role of AMF in supporting the survival of dominant plant species within the Aradie and Zengena forests. Both forests maintain viable AMF populations, though Zengena forest shows higher spore density and morphotype diversity (15 types) compared to Aradie (6 types).The genus *Acaulospora* is the most widespread and resilient fungal partner, appearing in 100% of sampled plants in Zengena forest and over 70% in Aradie forest. Specific plant species like *Cupressus lusitanica* and *Acacia abyssinica* act as “biodiversity hubs” for AMF, harboring the highest species richness. Since these fungi assist in nutrient uptake and stress tolerance, their presence is a major opportunity for forest regeneration in shrinking ecosystems for rehabilitation and restoration activities in Ethiopian Afromontane forests ecosystems. To address the continuous shrinking and degradation of these Afromontane ecosystems the following step by step practices are recommended: integrated restoration by using AMF inoculants, use of *Acacia abyssinica* and *Cupressus lusitanica* as primary species for land rehabilitation and restoration, minimize soil disturbance in the Aradie and Zengena forests and their surroundings, future research should compare these findings with wet-season data to understand how spore density fluctuates, ensuring that restoration efforts are timed for maximum fungal activity and the use molecular approaches in species identification.
