# Supplementary material for: Arbuscular mycorrhizal fungi spore density and species diversity varied within plants species and between dry Afromontane forests in northwestern Ethiopia
Source: PLoS One. 2026 Sep 3;21(9):e0329429. doi: 10.1371/journal.pone.0329429 (PMC13541138; doi:10.1371/journal.pone.0329429)
Supplement: Supplementary file 1 [file pone.0329429.s002.pdf]

The manuscript “Arbuscular mycorrhizal fungi spore density and species diversity varied within pants species and between dry Afromontane forests in northwestern Ethiopia” provides an interesting study, which is timely and relevant to the current scenario. However, few points need to be addressed before considering it suitable for acceptance to this esteemed journal. These are-

1. Page 9, line no. 75-76….”In their study these authors revealed a strong negative correlation between AMF spore density and an altitude.”….please rewrite the sentence.
2. Please check the spacing in between words, also in between two sentences throughout the manuscript. At several position, spacing are missing.
3. Page 9…line no. 80-84…”These AMF inoculations can improve seedling establishment and success rate due to their role in nutrient and water absorption and translocation, help the seedlings share growth limiting plant nutrients via common mycorrhizal 82 3 83 networks[15], help seedlings to tolerate abiotic stresses (drought, salinity, acidity) and biotic stresses (pathogens, competitions for nutrients and water)[10,15].”… please rewrite the sentence.
4. Page 10, line no. 85-97………no need to give those previous study in details, give the research gap and novelty of this study.
5. Introduction section needs to be shorten and focused to improve the clarity of this study.
6. It would be better to give the study location through location map.
7. Also, give the plant species authentication numbers in table 1.
8. Page 15….”Enumeration of AMF spores from each subsample was carried out under stereomicroscope at x40 magnification according to the methods and procedures described in the International Culture Collection of Vesicular-Arbuscular Mycorrhizal Fungi (INVAM), (http://invam.wu.edu/)” give full stop at the end of the sentence.
9. Page 16, line no. 207…remove the underline from the full stop.
10. 4.3 and 4.4 sections lacks critical discussion.
11. Conclusion section is very weak. Please improve
12. Include the limitation and future perspective of this study.
13. Figure 1 A and 1B…..Y axis title should be shorten, and X axis plant species name should be made uniform for both.
14. Please shorten the gap of x axis in between two forests bar.
