# Supplementary material for: Arbuscular mycorrhizal fungi spore density and species diversity varied within plants species and between dry Afromontane forests in northwestern Ethiopia
Source: PLoS One. 2026 Sep 3;21(9):e0329429. doi: 10.1371/journal.pone.0329429 (PMC13541138; doi:10.1371/journal.pone.0329429)
Supplement: Supplementary file 2 [file pone.0329429.s001.docx]

In order to be considered for publication, this manuscript's research paper requires some modifications and enhancements. Comments for revision are as follows:

**General Assessment**

The manuscript entitled “Arbuscular mycorrhizal fungi spore density and species diversity varied within plant species and between dry Afromontane forests in northwestern Ethiopia” addresses an important and underexplored topic related to soil microbial diversity and forest ecosystem restoration. The study is well designed, data are clearly presented, and the findings contribute useful baseline information for dry Afromontane forests in Ethiopia. Overall, the manuscript is suitable for publication after moderate revisions as outlined below.

**Major and Minor Comments are imposed to do corrections at author level**

**1. Title and Language Clarity**

The title is informative but contains minor grammatical issues (for example, “pants species” should be corrected to “plant species”). Overall language is understandable, but moderate English editing is required to improve grammar, sentence structure, and consistency throughout the manuscript.

**2. Abstract**

The abstract clearly summarizes the objectives, methods, and key findings. Minor corrections are suggested to improve readability and correct spacing issues, especially around scientific names.

**3. Introduction**

The introduction is comprehensive and well referenced. However, the research gap and novelty could be highlighted more explicitly in the final paragraph to strengthen the justification of the study.

**4. Study Design and Sampling**

The sampling strategy and site description are clearly explained and appropriate for the objectives. A brief justification for the number of plots and sampling period would further strengthen methodological rigor.

**5. Soil Physicochemical Analysis**

Standard and accepted methods were used. It is suggested to briefly discuss how soil parameters may have influenced AMF spore density differences between the two forests.

**6. AMF Identification and Enumeration**

The use of wet sieving, decantation, and morphological identification is appropriate. However, limitations of morphological identification without molecular confirmation should be briefly acknowledged in the discussion.

**7. Statistical Analysis**

The statistical methods used (ANOVA and Tukey’s test) are appropriate. Please ensure uniform reporting of significance levels (for example, p < 0.05) throughout the text and figure captions.

**8. Results Presentation**

Results are clearly presented with appropriate tables and figures. Minor formatting issues exist in tables (alignment and consistency in species names) and should be corrected.

**9. Discussion**

The discussion is detailed and well linked with previous studies. Some sections are repetitive; concise restructuring would improve clarity and focus. Practical implications for forest restoration using AMF could be emphasized further.

**10. Conclusion and Implications**

The conclusions are supported by the results. Adding a short paragraph on future research directions, such as molecular AMF diversity and inoculation trials, would enhance the manuscript’s impact.

**Final Recommendation**

**Recommendation: Accept with moderate revisions.**

The manuscript presents scientifically sound data and meaningful ecological insights. The required revisions are mainly related to language polishing, minor methodological clarifications, and formatting improvements, and do not affect the core scientific merit of the study. After addressing the above comments, the manuscript will be suitable for publication.
