# Supplementary material for: Arbuscular mycorrhizal fungi spore density and species diversity varied within plants species and between dry Afromontane forests in northwestern Ethiopia
Source: PLoS One. 2026 Sep 3;21(9):e0329429. doi: 10.1371/journal.pone.0329429 (PMC13541138; doi:10.1371/journal.pone.0329429)
Supplement: Supplementary file 3 [file pone.0329429.s004.docx]

**19.03.2026**

**Point by point response to the reviewers**

PONE-D-25-33914

Title: Arbuscular mycorrhizal fungi spore density and species diversity varied within plants species and between dry Afromontane forests in northwestern Ethiopia

**Dear Editors and reviewers**

We, the authors of this manuscript, thank you for giving chance to revise and improve our manuscript. We highly acknowledge and appreciate your constructive comments, suggestions and time invested to improve the quality and readability of our manuscript. We learn a lot from your views and way you see our manuscript. Truly speaking, all comments and suggestion forwarded are complementary. There are not contradicting comments from the reviewers. We addressed the general and specific comments and incorporated into the manuscripts and improved it significantly. We finally made a revision on the manuscript according to your general and specific comments and suggestions. Here below, you will find our point by point response.

With due respect

Belay Berza Beyene (Ph.D)

The corresponding author

**March 19.2026**

**Point by point response to the reviewers**

**Reviewer #1:**

**Reviewer comment**: General Assessment: The manuscript investigates AMF spore density and diversity in two Ethiopian Afromontane forests. The topic is relevant to ecology, biodiversity, and restoration, and the study contributes useful data from a region where such research is limited.

**Authors’ response:** Thanks very much for your positive comments and understanding. Your views encouraged us to improve our manuscript.

**Comment 1**:. Language and Grammar: There are numerous grammatical and typographical errors starting from the title through to the conclusion (check lines 1, 9-31, 43-45). I recommend that the manuscript undergo professional English editing.

**Authors’ response**: Thanks for your comments. We have checked all typo and spelling errors. Moreover, English language editing was also made.

**comment 2**:. Methods and Reproducibility: The Materials and Methods section lacks clarity on sample replication, randomization, and statistical assumptions. For example:
Line 138-141: “Five to ten dominant plant species were considered from each plot” is vague. Specify the exact number and criteria for selection.
Line 153–156: Clarify how composite samples were created and how many replicates per species per forest were analyzed. Add soil classification for both sites (lines 116–135).

**Authors’ response: Thanks for your very nice comments. The views are carefully addressed as required and the soil classification of the forests is also included, (Lines. )**

**comment 3:** Statistical Analysis: The authors reported ANOVA and Tukey’s HSD but did not justify statistical assumptions (normality, homogeneity of variance) or provide measures of variation (SD/SE).

**Authors’ response**: Thanks. We have already done those assumption but we did not include in the manuscript. We got the chance to incorporate in the manuscript (line……)

**comment 4**. Results Presentation: The results are clear but mostly descriptive without statistical backing (lines 254 – 261). Explanations about potential causes (e.g., soil type, altitude, and disturbance) appear in the Results (lines 266–268) and should be moved to the Discussion.

**Authors’ response: Thanks this is very good comment and suggestion. We revisited the result section and incorporated the statistical significances. We have also moved the discussion into the discussion section.**

**comment 5**. The discussion repeats results excessively and lists numerous studies without deeper analysis (Lines 323–338 and 369–396). Authors should synthesize findings into clear messages: (a) what environmental factors drive differences between the two forests? (b) how AMF-host specificity is expressed, and (c) what are the implications for restoration practices?.

**Authors’ response: Many thanks. This is a core point of the work. This is all about the whole work. We appreciate your comments and accordingly incorporated in the discussions.**

**Comment 6**. The conclusion currently reads more like a summary of the results. It should be revised to emphasize the novelty of the findings, acknowledge study limitations, and outline their practical applications.

**Authors’ response: Thanks for your suggestion. The conclusion section is significantly improved. We have incorporated the limitation and practical implications.**

**Comment 7.** Several references are outdated (e.g., Schenck 1990; Gerdemann & Nicolson 1963; Walkley & Black 1934) and should be updated with more recent studies. In addition, reference formatting should be standardized by italicizing species names, and consolidating redundant citations (e.g., Birhane et al. [6,7,23] cited multiple times for similar points).

**Authors’ response: Thanks again. Here the point is correct. However, those updated references are the original methods still in use in AMF research. We have made serious check in the references and italized the scientific names. We repeatedly cited Birhane et al because his work is the only work we obtained in the north and northwest Ethiopia.**

**comment 8**. Abstract (lines 9–31): Correct multiple typos, spacing issues, and species name inconsistencies.

**Authors’ response: Thanks. All typo, spelling and spacing issues are addressed.**

**Comment	9**. Introduction: Literature review is too long and repetitive. Condense into a focused summary.

**Authors’ response: Thanks. Done as suggested.**

**Comment 10.** Figures/Tables: Ensure captions and units are clear. For example: “AMF Spore Density" ( 100 g⁻¹ soil).”

**Authors’ response: Thanks. Done as required.**

**Comment 11**. Terminology: Use consistent species spelling throughout (e.g., Cupressus lusitanica not Cupressu lucitanica; Olea europaea not Olea europea).

**Authors’ response: Thank you very much. We accept your comments and suggestions and incorporated in to the manuscript.**

**Reviewer #2:** 
The manuscript entitled “Arbuscular mycorrhizal fungi spore density and species diversity varied within plant species and between dry Afromontane forests in northwestern Ethiopia” addresses an important and underexplored topic related to soil microbial diversity and forest ecosystem restoration. The study is well designed, data are clearly presented, and the findings contribute useful baseline information for dry Afromontane forests in Ethiopia. Overall, the manuscript is suitable for publication after moderate revisions as outlined below.

**Authors’ response: Many thanks for your encouraging and positive views which acted as a spring board to work.**

**comment 1**. The title is informative but contains minor grammatical issues (for example, “pants species” should be corrected to “plant species”). Overall language is understandable, but moderate English editing is required to improve grammar, sentence structure, and consistency throughout the manuscript.

**Authors’ response: Thanks for your comments and suggestions. We addressed grammatical, typo, spacing and sentence structure issues. We have also made English language editing throughout the manuscript.**

**Comment	2**. The abstract clearly summarizes the objectives, methods, and key findings. Minor corrections are suggested to improve readability and correct spacing issues, especially around scientific names.

**Authors’ response: Thanks for your comments. We addressed as required.**

**Comment 3.** The introduction is comprehensive and well referenced. However, the research gap and novelty could be highlighted more explicitly in the final paragraph to strengthen the justification of the study.

**Authors’ response: Thanks. We have incorporated research gaps, hypothesis and novelty of the work.**

**Comment	4**. The sampling strategy and site description are clearly explained and appropriate for the objectives. A brief justification for the number of plots and sampling period would further strengthen methodological rigor.

**Authors’ response: Thank you for your suggestion. We refined the methodology section carefully. Clear justification are incorporated.**

**Comment	5**. Standard and accepted methods were used. It is suggested to briefly discuss how soil parameters may have influenced AMF spore density differences between the two forests.

**Authors’ response: Thanks. It is incorporated as requested.**

**Comment	6**. The use of wet sieving, decantation, and morphological identification is appropriate. However, limitations of morphological identification without molecular confirmation should be briefly acknowledged in the discussion.

**Authors’ response: Thanks again for this important point. We accept your suggestion and included (lines 470-480).**

**Comment 7**. The statistical methods used (ANOVA and Tukey’s test) are appropriate. Please ensure uniform reporting of significance levels (for example, p < 0.05) throughout the text and figure captions.

**Authors’ response: Thanks. Your comments and suggestions are addressed.**

**Comment 8**. Results are clearly presented with appropriate tables and figures. Minor formatting issues exist in tables (alignment and consistency in species names) and should be corrected.

**Authors’ response: Thank you very much. We have made serious formatting in the tables and figures.**

**Comment 9**. The discussion is detailed and well linked with previous studies. Some sections are repetitive; concise restructuring would improve clarity and focus. Practical implications for forest restoration using AMF could be emphasized further.

**Authors’ response: Thanks very much. We really appreciate your views. We removed or deleted repetitions. The practical implication for biodiversity conservation, rehabilitation and restoration are incorporated.**

**Comment 10**. The conclusions are supported by the results. Adding a short paragraph on future research directions, such as molecular AMF diversity and inoculation trials, would enhance the manuscript’s impact.

**Authors’ response: Many thanks. The conclusion section is significantly improved. (Lines 488- 503)**

**Reviewer #3:**

The manuscript “Arbuscular mycorrhizal fungi spore density and species diversity varied within pants species and between dry Afromontane forests in northwestern Ethiopia” provides an interesting study, which is timely and relevant to the current scenario.

**Authors’ response**: **Many thanks for your encouraging and positive views which acted as a spring board to work.**

**Comment 1**: Page 9, line no. 75-76….”In their study these authors revealed a strong negative correlation between AMF spore density and an altitude.”….please rewrite the sentence.

**Authors’ response: Thanks. Rewritten. Done as needed.**

**Comment 2**. Please check the spacing in between words, also in between two sentences throughout the manuscript. At several position, spacing are missing.

**Authors’ response: Thank again. Typo, spacing, grammatical and English editing has been made to improve the manuscript.**

**Comment 3**. Page 9…line no. 80-84…”These AMF inoculations can improve seedling establishment and success rate due to their role in nutrient and water absorption and translocation, help the seedlings share growth limiting plant nutrients via common mycorrhizal 82 3 83 networks[15], help seedlings to tolerate abiotic stresses (drought, salinity, acidity) and biotic stresses (pathogens, competitions for nutrients and water)[10,15].”… please rewrite the sentence.

**Authors’ response: Thanks. Done as required.**

**Comment 4**. Page 10, line no. 85-97………no need to give those previous study in details, give the research gap and novelty of this study.

**Authors’ response: Thanks. Done**

**Comment 5**. Introduction section needs to be shorten and focused to improve the clarity of this study.

**Authors’ response: Many thanks for your comments. Done.**

**Comment 6**. It would be better to give the study location through location map.

**Authors’ response: Thanks. Map of the study areas is incorporated as Fig.1 (line…121)**

**Comment	7**. Page 15….”Enumeration of AMF spores from each subsample was carried out under stereomicroscope at x40 magnification according to the methods and procedures described in the International Culture Collection of Vesicular-Arbuscular Mycorrhizal Fungi (INVAM), ([http://invam.wu.edu/)](http://invam.wu.edu/))” give full stop at the end of the sentence.

**Authors’ response: Thanks. It is done.**

**Comment 8**. Page 16, line no. 207…remove the underline from the full stop.

**Authors’ response: Thanks. Done**

**Comment	9** . 4.3 and 4.4 sections lacks critical discussion.

**Authors’ response: Thanks. The discussion section is significantly improved by incorporating all your positive suggestions.**

**Comment 10**. Conclusion section is very weak. Please improve

**Authors’ response: Thanks. This section is significantly improved (line…..)**

**Comment	11**. Include the limitation and future perspective of this study.

**Authors’ response: Thanks for your views. We have incorporated in the discussion and conclusion section.**

**Comment 12**. Figure 1 A and 1B…..Y axis title should be shorten, and X axis plant species name should be made uniform for both.

**Authors’ response: Thanks. Corrections are made.**

**Comment 13**. Please shorten the gap of x axis in between two forests bar.

**Authors’ response: Thanks. Done as required.**

**Reviewer #4:**

**Comment 1**: The specific research questions are not explicitly stated in question form. While the objectives imply what the study aims to address, the manuscript would benefit from stating them directly.

**Authors’ response: Thank you for your encouraging comments and suggestions. We took your suggestion and incorporated accordingly.**

**Comment	2**. The manuscript does not clearly state a hypothesis. A well-defined hypothesis would strengthen the scientific rigor of the study and help guide the reader's expectations.

**Authors’ response: Thanks. We incorporated the hypothesis (lines 112-114).**

**Comment	3**. The research gap is mentioned but should be sharpened to explicitly state what is new compared to previous AMF studies in Ethiopian Afromontane forests.

**Authors’ response: Thanks. We incorporated your comments.**

**Comment 4**. The sampling design needs clearer justification, including rationale for plot number, plot size, and selection of dominant plant species.

**Authors’ response: Thanks. We did as required.**

**Comment 5**. The pooling of soil samples should be justified, and its implications for variability and statistical power should be discussed.

**Authors’ response: Thanks again. We clarified the pooling issues.**

**Comment	6**. The statistical analysis section should specify the experimental unit, assumptions checked for ANOVA, and how normality and homogeneity of variance were addressed.

**Authors’ response: Thanks for very important point. We have incorporated in the manuscript (lines 231- 238).**

**Comment	7**. Methods for AMF identification should clarify how uncertain morphotypes (identified only to genus level) were handled in analyses

**Authors’ response: Many thanks. We addressed your points.**
