# Supplementary material for: Arbuscular mycorrhizal fungi spore density and species diversity varied within plants species and between dry Afromontane forests in northwestern Ethiopia
Source: PLoS One. 2026 Sep 3;21(9):e0329429. doi: 10.1371/journal.pone.0329429 (PMC13541138; doi:10.1371/journal.pone.0329429)
Supplement: Supplementary file 4 [file pone.0329429.s005.docx]

**10.07.2026**

Point by point response to the reviewers

Arbuscular mycorrhizal fungi spore density and species diversity varied within plants species and between dry Afromontane forests in northwestern Ethiopia

PONE-D-25-33914R1

Dear Dr. Erika Kothe

Academic Editor, Plosone, and Reviewers

Many thanks for your contribution in our career development and giving this golden opportunity to revise and improve our manuscript. We highly acknowledge and appreciate your critical reviews, inputs and time invested to improve the quality of our manuscript. We learned a lot from your review comments and suggestions. We accepted all your general and specific comments and suggestion with full recognition. We have also made revision to the manuscript as per your comments and suggestions. Here below, we forward the point by point response for reviewers’ comments and suggestions.

With due respect

Belay Berza Beyene (the submitting author)

**July 10.2026**

**Point by point response to the reviewers**

**Reviewer #1**

**Comment 1**;  Reviewer #3: Authors have addressed most of the comments satisfactorily. However, line no. 112-116 should be written in a better way to meet the standard of this journal.

**Response to reviewer: We highly appreciate your comments and suggestions. All your comments and suggestions are incorporated accordingly in the materials and method section.( lines 112-116)**
