# Supplementary material for: Arbuscular mycorrhizal fungi spore density and species diversity varied within plants species and between dry Afromontane forests in northwestern Ethiopia
Source: PLoS One. 2026 Sep 3;21(9):e0329429. doi: 10.1371/journal.pone.0329429 (PMC13541138; doi:10.1371/journal.pone.0329429)
Supplement: Peer review history

# Peer review: Decision Letter 0

**DOI:** 10.1371/journal.pone.0329429.r001

**Authors:** Debasis Mitra

4 Feb 2026

Dear Dr. Beyene,

Thank you for submitting your manuscript to PLOS ONE. After careful consideration, we feel that it has merit but does not fully meet PLOS ONE’s publication criteria as it currently stands. Therefore, we invite you to submit a revised version of the manuscript that addresses the points raised during the review process.

Please submit your revised manuscript by Mar 19 2026 11:59PM. If you will need more time than this to complete your revisions, please reply to this message or contact the journal office at plosone@plos.org. When you're ready to submit your revision, log on to https://www.editorialmanager.com/pone/ and select the 'Submissions Needing Revision' folder to locate your manuscript file.

- A letter that responds to each point raised by the academic editor and reviewer(s). You should upload this letter as a separate file labeled 'Response to Reviewers'.
- A marked-up copy of your manuscript that highlights changes made to the original version. You should upload this as a separate file labeled 'Revised Manuscript with Track Changes'.
- An unmarked version of your revised paper without tracked changes. You should upload this as a separate file labeled 'Manuscript'.

If you would like to make changes to your financial disclosure, please include your updated statement in your cover letter. Guidelines for resubmitting your figure files are available below the reviewer comments at the end of this letter.

If applicable, we recommend that you deposit your laboratory protocols in protocols.io to enhance the reproducibility of your results. Protocols.io assigns your protocol its own identifier (DOI) so that it can be cited independently in the future. For instructions see: https://journals.plos.org/plosone/s/submission-guidelines#loc-laboratory-protocols. Additionally, PLOS ONE offers an option for publishing peer-reviewed Lab Protocol articles, which describe protocols hosted on protocols.io. Read more information on sharing protocols at https://plos.org/protocols?utm_medium=editorial-email&utm_source=authorletters&utm_campaign=protocols.

We look forward to receiving your revised manuscript.

Kind regards,

Debasis Mitra

Academic Editor

PLOS One

Journal Requirements:

When submitting your revision, we need you to address these additional requirements.

1. Please ensure that your manuscript meets PLOS ONE's style requirements, including those for file naming. The PLOS ONE style templates can be found at

https://journals.plos.org/plosone/s/file?id=wjVg/PLOSOne_formatting_sample_main_body.pdf and

https://journals.plos.org/plosone/s/file?id=ba62/PLOSOne_formatting_sample_title_authors_affiliations.pdf

2. When completing the data availability statement of the submission form, you indicated that you will make your data available on acceptance. We strongly recommend all authors decide on a data sharing plan before acceptance, as the process can be lengthy and hold up publication timelines. Please note that, though access restrictions are acceptable now, your entire data will need to be made freely accessible if your manuscript is accepted for publication. This policy applies to all data except where public deposition would breach compliance with the protocol approved by your research ethics board. If you are unable to adhere to our open data policy, please kindly revise your statement to explain your reasoning and we will seek the editor's input on an exemption. Please be assured that, once you have provided your new statement, the assessment of your exemption will not hold up the peer review process.

3. If the reviewer comments include a recommendation to cite specific previously published works, please review and evaluate these publications to determine whether they are relevant and should be cited. There is no requirement to cite these works unless the editor has indicated otherwise.

[Note: HTML markup is below. Please do not edit.]

Reviewers' comments:

Reviewer's Responses to Questions

**Comments to the Author**

1. Is the manuscript technically sound, and do the data support the conclusions?

Reviewer #1: Partly

Reviewer #2: Yes

Reviewer #3: Yes

Reviewer #4: No

**********

2. Has the statistical analysis been performed appropriately and rigorously?

Reviewer #1: Yes

Reviewer #2: Yes

Reviewer #3: Yes

Reviewer #4: No

**********

3. Have the authors made all data underlying the findings in their manuscript fully available?

The PLOS Data policy

Reviewer #1: Yes

Reviewer #2: Yes

Reviewer #3: Yes

Reviewer #4: Yes

**********

4. Is the manuscript presented in an intelligible fashion and written in standard English?

Reviewer #1: Yes

Reviewer #2: Yes

Reviewer #3: Yes

Reviewer #4: No

**********

Reviewer #1: General Assessment: The manuscript investigates AMF spore density and diversity in two Ethiopian Afromontane forests. The topic is relevant to ecology, biodiversity, and restoration, and the study contributes useful data from a region where such research is limited. However, the manuscript requires major revision before it is suitable for publication. Key issues include English language quality, methodological clarity, consistency in taxonomy, statistical rigor, and redundancy in presentation.

Major Comments:

1. Language and Grammar: There are numerous grammatical and typographical errors starting from the title through to the conclusion (check lines 1, 9-31, 43-45). I recommend that the manuscript undergo professional English editing.

2. Methods and Reproducibility: The Materials and Methods section lacks clarity on sample replication, randomization, and statistical assumptions. For example:

Line 138-141: “Five to ten dominant plant species were considered from each plot” is vague. Specify the exact number and criteria for selection.

Line 153–156: Clarify how composite samples were created and how many replicates per species per forest were analyzed.

Add soil classification for both sites (lines 116–135).

3. Statistical Analysis: The authors reported ANOVA and Tukey’s HSD but did not justify statistical assumptions (normality, homogeneity of variance) or provide measures of variation (SD/SE).

4. Results Presentation: The results are clear but mostly descriptive without statistical backing (lines 254 – 261). Explanations about potential causes (e.g., soil type, altitude, disturbance) appear in the Results (lines 266–268) and should be moved to the Discussion.

5. Discussion and Synthesis: The discussion repeats results excessively and lists numerous studies without deeper analysis (Lines 323–338 and 369–396). Authors should synthesize findings into clear messages: (a) what environmental factors drive differences between the two forests? (b) how AMF-host specificity is expressed, and (c) what are the implications for restoration practices?.

6. Conclusion: The conclusion currently reads more like a summary of the results. It should be revised to emphasize the novelty of the findings, acknowledge study limitations, and outline their practical applications.

7. References: Several references are outdated (e.g., Schenck 1990; Gerdemann & Nicolson 1963; Walkley & Black 1934) and should be updated with more recent studies. In addition, reference formatting should be standardized by italicizing species names, and consolidating redundant citations (e.g., Birhane et al. [6,7,23] cited multiple times for similar points).

Minor Comments:

1. Abstract (lines 9–31): Correct multiple typos, spacing issues, and species name inconsistencies.

2. Introduction: Literature review is too long and repetitive. Condense into a focused summary.

3. Figures/Tables: Ensure captions and units are clear. For example: “AMF Spore Density" ( 100 g⁻¹ soil).”

4. Terminology: Use consistent species spelling throughout (e.g., Cupressus lusitanica not Cupressu lucitanica; Olea europaea not Olea europea).

Reviewer #2: In order to be considered for publication, this manuscript's research paper requires some modifications and enhancements. Comments for revision are as follows:

General Assessment

The manuscript entitled “Arbuscular mycorrhizal fungi spore density and species diversity varied within plant species and between dry Afromontane forests in northwestern Ethiopia” addresses an important and underexplored topic related to soil microbial diversity and forest ecosystem restoration. The study is well designed, data are clearly presented, and the findings contribute useful baseline information for dry Afromontane forests in Ethiopia. Overall, the manuscript is suitable for publication after moderate revisions as outlined below.

Major and Minor Comments are imposed to do corrections at author level

1. Title and Language Clarity

The title is informative but contains minor grammatical issues (for example, “pants species” should be corrected to “plant species”). Overall language is understandable, but moderate English editing is required to improve grammar, sentence structure, and consistency throughout the manuscript.

2. Abstract

The abstract clearly summarizes the objectives, methods, and key findings. Minor corrections are suggested to improve readability and correct spacing issues, especially around scientific names.

3. Introduction

The introduction is comprehensive and well referenced. However, the research gap and novelty could be highlighted more explicitly in the final paragraph to strengthen the justification of the study.

4. Study Design and Sampling

The sampling strategy and site description are clearly explained and appropriate for the objectives. A brief justification for the number of plots and sampling period would further strengthen methodological rigor.

5. Soil Physicochemical Analysis

Standard and accepted methods were used. It is suggested to briefly discuss how soil parameters may have influenced AMF spore density differences between the two forests.

6. AMF Identification and Enumeration

The use of wet sieving, decantation, and morphological identification is appropriate. However, limitations of morphological identification without molecular confirmation should be briefly acknowledged in the discussion.

7. Statistical Analysis

The statistical methods used (ANOVA and Tukey’s test) are appropriate. Please ensure uniform reporting of significance levels (for example, p < 0.05) throughout the text and figure captions.

8. Results Presentation

Results are clearly presented with appropriate tables and figures. Minor formatting issues exist in tables (alignment and consistency in species names) and should be corrected.

9. Discussion

The discussion is detailed and well linked with previous studies. Some sections are repetitive; concise restructuring would improve clarity and focus. Practical implications for forest restoration using AMF could be emphasized further.

10. Conclusion and Implications

The conclusions are supported by the results. Adding a short paragraph on future research directions, such as molecular AMF diversity and inoculation trials, would enhance the manuscript’s impact.

Final Recommendation

Recommendation: Accept with moderate revisions.

The manuscript presents scientifically sound data and meaningful ecological insights. The required revisions are mainly related to language polishing, minor methodological clarifications, and formatting improvements, and do not affect the core scientific merit of the study. After addressing the above comments, the manuscript will be suitable for publication.

Reviewer #3: The manuscript “Arbuscular mycorrhizal fungi spore density and species diversity varied within pants species and between dry Afromontane forests in northwestern Ethiopia” provides an interesting study, which is timely and relevant to the current scenario. However, few points need to be addressed before considering it suitable for acceptance to this esteemed journal. These are-

1. Page 9, line no. 75-76….”In their study these authors revealed a strong negative correlation between AMF spore density and an altitude.”….please rewrite the sentence.

2. Please check the spacing in between words, also in between two sentences throughout the manuscript. At several position, spacing are missing.

3. Page 9…line no. 80-84…”These AMF inoculations can improve seedling establishment and success rate due to their role in nutrient and water absorption and translocation, help the seedlings share growth limiting plant nutrients via common mycorrhizal 82 3 83 networks[15], help seedlings to tolerate abiotic stresses (drought, salinity, acidity) and biotic stresses (pathogens, competitions for nutrients and water)[10,15].”… please rewrite the sentence.

4. Page 10, line no. 85-97………no need to give those previous study in details, give the research gap and novelty of this study.

5. Introduction section needs to be shorten and focused to improve the clarity of this study.

6. It would be better to give the study location through location map.

7. Also, give the plant species authentication numbers in table 1.

8. Page 15….”Enumeration of AMF spores from each subsample was carried out under stereomicroscope at x40 magnification according to the methods and procedures described in the International Culture Collection of Vesicular-Arbuscular Mycorrhizal Fungi (INVAM), (http://invam.wu.edu/)” give full stop at the end of the sentence.

9. Page 16, line no. 207…remove the underline from the full stop.

10. 4.3 and 4.4 sections lacks critical discussion.

11. Conclusion section is very weak. Please improve

12. Include the limitation and future perspective of this study.

13. Figure 1 A and 1B…..Y axis title should be shorten, and X axis plant species name should be made uniform for both.

14. Please shorten the gap of x axis in between two forests bar.

Reviewer #4: The manuscript explores the ‘Arbuscular mycorrhizal fungi spore density and species diversity varied within pants species and between dry Afromontane forests in northwestern Ethiopia’. However, there are several critical comments and suggestions that can be made to improve the clarity and effectiveness of the manuscript.

1. The specific research questions are not explicitly stated in question form. While the objectives imply what the study aims to address, the manuscript would benefit from stating them directly.

2. The manuscript does not clearly state a hypothesis. A well-defined hypothesis would strengthen the scientific rigor of the study and help guide the reader's expectations.

3. The research gap is mentioned but should be sharpened to explicitly state what is new compared to previous AMF studies in Ethiopian Afromontane forests.

4. The sampling design needs clearer justification, including rationale for plot number, plot size, and selection of dominant plant species.

5. The pooling of soil samples should be justified, and its implications for variability and statistical power should be discussed.

6. The statistical analysis section should specify the experimental unit, assumptions checked for ANOVA, and how normality and homogeneity of variance were addressed.

7. Methods for AMF identification should clarify how uncertain morphotypes (identified only to genus level) were handled in analyses.

8. The presentation of species richness and diversity indices could be strengthened by including additional diversity metrics (e.g., Shannon or Simpson indices) or by justifying their exclusion.

**********

what does this mean?). If published, this will include your full peer review and any attached files.

If you choose “no”, your identity will remain anonymous but your review may still be made public.

**Do you want your identity to be public for this peer review?**  For information about this choice, including consent withdrawal, please see our Privacy Policy

Reviewer #1: No

Reviewer #2: No

Reviewer #3: **Yes:**  Dr. Kartik Chandra Guchhait, Post-doctoral Fellow, IIT Kharagpur, WB, India

Reviewer #4: **Yes:**  Dr. DASARI SREEKANTH

**********

[NOTE: If reviewer comments were submitted as an attachment file, they will be attached to this email and accessible via the submission site. Please log into your account, locate the manuscript record, and check for the action link "View Attachments". If this link does not appear, there are no attachment files.]

To ensure your figures meet our technical requirements, please review our figure guidelines: https://journals.plos.org/plosone/s/figures

You may also use PLOS’s free figure tool, NAAS, to help you prepare publication quality figures: https://journals.plos.org/plosone/s/figures#loc-tools-for-figure-preparation.

NAAS will assess whether your figures meet our technical requirements by comparing each figure against our figure specifications.

Attachment Submitted filename: plos one.docx

Attachment Submitted filename: Comment.pdf

# Author comment: Author response to Decision Letter 1

**DOI:** 10.1371/journal.pone.0329429.r002

20 Mar 2026

19.03.2026

Point by point response to the reviewers

PONE-D-25-33914

Title: Arbuscular mycorrhizal fungi spore density and species diversity varied within plants species and between dry Afromontane forests in northwestern Ethiopia

Dear Editors and reviewers

We, the authors of this manuscript, thank you for giving chance to revise and improve our manuscript. We highly acknowledge and appreciate your constructive comments, suggestions and time invested to improve the quality and readability of our manuscript. We learn a lot from your views and way you see our manuscript. Truly speaking, all comments and suggestion forwarded are complementary. There are not contradicting comments from the reviewers. We addressed the general and specific comments and incorporated into the manuscripts and improved it significantly. We finally made a revision on the manuscript according to your general and specific comments and suggestions. Here below, you will find our point by point response.

With due respect

Belay Berza Beyene (Ph.D)

The corresponding author

March 19.2026

Point by point response to the reviewers

Reviewer #1:

Reviewer comment: General Assessment: The manuscript investigates AMF spore density and diversity in two Ethiopian Afromontane forests. The topic is relevant to ecology, biodiversity, and restoration, and the study contributes useful data from a region where such research is limited.

Authors’ response: Thanks very much for your positive comments and understanding. Your views encouraged us to improve our manuscript.

Comment 1:. Language and Grammar: There are numerous grammatical and typographical errors starting from the title through to the conclusion (check lines 1, 9-31, 43-45). I recommend that the manuscript undergo professional English editing.

Authors’ response: Thanks for your comments. We have checked all typo and spelling errors. Moreover, English language editing was also made.

comment 2:. Methods and Reproducibility: The Materials and Methods section lacks clarity on sample replication, randomization, and statistical assumptions. For example:

Line 138-141: “Five to ten dominant plant species were considered from each plot” is vague. Specify the exact number and criteria for selection.

Line 153–156: Clarify how composite samples were created and how many replicates per species per forest were analyzed. Add soil classification for both sites (lines 116–135).

Authors’ response: Thanks for your very nice comments. The views are carefully addressed as required and the soil classification of the forests is also included, (Lines. )

comment 3: Statistical Analysis: The authors reported ANOVA and Tukey’s HSD but did not justify statistical assumptions (normality, homogeneity of variance) or provide measures of variation (SD/SE).

Authors’ response: Thanks. We have already done those assumption but we did not include in the manuscript. We got the chance to incorporate in the manuscript (line……)

comment 4. Results Presentation: The results are clear but mostly descriptive without statistical backing (lines 254 – 261). Explanations about potential causes (e.g., soil type, altitude, and disturbance) appear in the Results (lines 266–268) and should be moved to the Discussion.

Authors’ response: Thanks this is very good comment and suggestion. We revisited the result section and incorporated the statistical significances. We have also moved the discussion into the discussion section.

comment 5. The discussion repeats results excessively and lists numerous studies without deeper analysis (Lines 323–338 and 369–396). Authors should synthesize findings into clear messages: (a) what environmental factors drive differences between the two forests? (b) how AMF-host specificity is expressed, and (c) what are the implications for restoration practices?.

Authors’ response: Many thanks. This is a core point of the work. This is all about the whole work. We appreciate your comments and accordingly incorporated in the discussions.

Comment 6. The conclusion currently reads more like a summary of the results. It should be revised to emphasize the novelty of the findings, acknowledge study limitations, and outline their practical applications.

Authors’ response: Thanks for your suggestion. The conclusion section is significantly improved. We have incorporated the limitation and practical implications.

Comment 7. Several references are outdated (e.g., Schenck 1990; Gerdemann & Nicolson 1963; Walkley & Black 1934) and should be updated with more recent studies. In addition, reference formatting should be standardized by italicizing species names, and consolidating redundant citations (e.g., Birhane et al. [6,7,23] cited multiple times for similar points).

Authors’ response: Thanks again. Here the point is correct. However, those updated references are the original methods still in use in AMF research. We have made serious check in the references and italized the scientific names. We repeatedly cited Birhane et al because his work is the only work we obtained in the north and northwest Ethiopia.

comment 8. Abstract (lines 9–31): Correct multiple typos, spacing issues, and species name inconsistencies.

Authors’ response: Thanks. All typo, spelling and spacing issues are addressed.

Comment 9. Introduction: Literature review is too long and repetitive. Condense into a focused summary.

Authors’ response: Thanks. Done as suggested.

Comment 10. Figures/Tables: Ensure captions and units are clear. For example: “AMF Spore Density" ( 100 g⁻¹ soil).”

Authors’ response: Thanks. Done as required.

Comment 11. Terminology: Use consistent species spelling throughout (e.g., Cupressus lusitanica not Cupressu lucitanica; Olea europaea not Olea europea).

Authors’ response: Thank you very much. We accept your comments and suggestions and incorporated in to the manuscript.

Reviewer #2:

The manuscript entitled “Arbuscular mycorrhizal fungi spore density and species diversity varied within plant species and between dry Afromontane forests in northwestern Ethiopia” addresses an important and underexplored topic related to soil microbial diversity and forest ecosystem restoration. The study is well designed, data are clearly presented, and the findings contribute useful baseline information for dry Afromontane forests in Ethiopia. Overall, the manuscript is suitable for publication after moderate revisions as outlined below.

Authors’ response: Many thanks for your encouraging and positive views which acted as a spring board to work.

comment 1. The title is informative but contains minor grammatical issues (for example, “pants species” should be corrected to “plant species”). Overall language is understandable, but moderate English editing is required to improve grammar, sentence structure, and consistency throughout the manuscript.

Authors’ response: Thanks for your comments and suggestions. We addressed grammatical, typo, spacing and sentence structure issues. We have also made English language editing throughout the manuscript.

Comment 2. The abstract clearly summarizes the objectives, methods, and key findings. Minor corrections are suggested to improve readability and correct spacing issues, especially around scientific names.

Authors’ response: Thanks for your comments. We addressed as required.

Comment 3. The introduction is comprehensive and well referenced. However, the research gap and novelty could be highlighted more explicitly in the final paragraph to strengthen the justification of the study.

Authors’ response: Thanks. We have incorporated research gaps, hypothesis and novelty of the work.

Comment 4. The sampling strategy and site description are clearly explained and appropriate for the objectives. A brief justification for the number of plots and sampling period would further strengthen methodological rigor.

Authors’ response: Thank you for your suggestion. We refined the methodology section carefully. Clear justification are incorporated.

Comment 5. Standard and accepted methods were used. It is suggested to briefly discuss how soil parameters may have influenced AMF spore density differences between the two forests.

Authors’ response: Thanks. It is incorporated as requested.

Comment 6. The use of wet sieving, decantation, and morphological identification is appropriate. However, limitations of morphological identification without molecular confirmation should be briefly acknowledged in the discussion.

Authors’ response: Thanks again for this important point. We accept your suggestion and included (lines 470-480).

Comment 7. The statistical methods used (ANOVA and Tukey’s test) are appropriate. Please ensure uniform reporting of significance levels (for example, p < 0.05) throughout the text and figure captions.

Authors’ response: Thanks. Your comments and suggestions are addressed.

Comment 8. Results are clearly presented with appropriate tables and figures. Minor formatting issues exist in tables (alignment and consistency in species names) and should be corrected.

Authors’ response: Thank you very much. We have made serious formatting in the tables and figures.

Comment 9. The discussion is detailed and well linked with previous studies. Some sections are repetitive; concise restructuring would improve clarity and focus. Practical implications for forest restoration using AMF could be emphasized further.

Authors’ response: Thanks very much. We really appreciate your views. We removed or deleted repetitions. The practical implication for biodiversity conservation, rehabilitation and restoration are incorporated.

Comment 10. The conclusions are supported by the results. Adding a short paragraph on future research directions, such as molecular AMF diversity and inoculation trials, would enhance the manuscript’s impact.

Authors’ response: Many thanks. The conclusion section is significantly improved. (Lines 488- 503)

Reviewer #3:

The manuscript “Arbuscular mycorrhizal fungi spore density and species diversity varied within pants species and between dry Afromontane forests in northwestern Ethiopia” provides an interesting study, which is timely and relevant to the current scenario.

Authors’ response: Many thanks for your encouraging and positive views which acted as a spring board to work.

Comment 1: Page 9, line no. 75-76….”In their study these authors revealed a strong negative correlation between AMF spore density and an altitude.”….please rewrite the sentence.

Authors’ response: Thanks. Rewritten. Done as needed.

Comment 2. Please check the spacing in between words, also in between two sentences throughout the manuscript. At several position, spacing are missing.

Authors’ response: Thank again. Typo, spacing, grammatical and English editing has been made to improve the manuscript.

Comment 3. Page 9…line no. 80-84…”These AMF inoculations can improve seedling establishment and success rate due to their role in nutrient and water absorption and translocation, help the seedlings share growth limiting plant nutrients via common mycorrhizal 82 3 83 networks[15], help seedlings to tolerate abiotic stresses (drought, salinity, acidity) and biotic stresses (pathogens, competitions for nutrients and water)[10,15].”… please rewrite the sentence.

Authors’ response: Thanks. Done as required.

Comment 4. Page 10, line no. 85-97………no need to give those previous study in details, give the research gap and novelty of this study.

Authors’ response: Thanks. Done

Comment 5. Introduction section needs to be shorten and focused to improve the clarity of this study.

Authors’ response: Many thanks for your comments. Done.

Comment 6. It would be better to give the study location through location map.

Authors’ response: Thanks. Map of the study areas is incorporated as Fig.1 (line…121)

Comment 7. Page 15….”Enumeration of AMF spores from each subsample was carried out under stereomicroscope at x40 magnification according to the methods and procedures described in the International Culture Collection of Vesicular-Arbuscular Mycorrhizal Fungi (INVAM), (http://invam.wu.edu/)” give full stop at the end of the sentence.

Authors’ response: Thanks. It is done.

Comment 8. Page 16, line no. 207…remove the underline from the full stop.

Authors’ response: Thanks. Done

Comment 9 . 4.3 and 4.4 sections lacks critical discussion.

Authors’ response: Thanks. The discussion section is significantly improved by incorporating all your positive suggestions.

Comment 10. Conclusion section is very weak. Please improve

Authors’ response: Thanks. This section is significantly improved (line…..)

Comment 11. Include the limitation and future perspective of this study.

Authors’ response: Thanks for your views. We have incorporated in the discussion and conclusion section.

Comment 12. Figure 1 A and 1B…..Y axis title should be shorten, and X axis plant species name should be made uniform for both.

Authors’ response: Thanks. Corrections are made.

Comment 13. Please shorten the gap of x axis in between two forests bar.

Authors’ response: Thanks. Done as required.

Reviewer #4:

Comment 1: The specific research questions are not explicitly stated in question form. While the objectives imply what the study aims to address, the manuscript would benefit from stating them directly.

Authors’ response: Thank you for your encouraging comments and suggestions. We took your suggestion and incorporated accordingly.

Comment 2. The manuscript does not clearly state a hypothesis. A well-defined hypothesis would strengthen the scientific rigor of the study and help guide the reader's expectations.

Authors’ response: Thanks. We incorporated the hypothesis (lines 112-114).

Comment 3. The research gap is mentioned but should be sharpened to explicitly state what is new compared to previous AMF studies in Ethiopian Afromontane forests.

Authors’ response: Thanks. We incorporated your comments.

Comment 4. The sampling design needs clearer justification, including rationale for plot number, plot size, and selection of dominant plant species.

Authors’ response: Thanks. We did as required.

Comment 5. The pooling of soil samples should be justified, and its implications for variability and statistical power should be discussed.

Authors’ response: Thanks again. We clarified the pooling issues.

Comment 6. The statistical analysis section should specify the experimental unit, assumptions checked for ANOVA, and how normality and homogeneity of variance were addressed.

Authors’ response: Thanks for very important point. We have incorporated in the manuscript (lines 231- 238).

Comment 7. Methods for AMF identification should clarify how uncertain morphotypes (identified only to genus level) were handled in analyses

Authors’ response: Many thanks. We addressed your points.

Attachment Submitted filename: Point by point response to reviewers.docx

# Peer review: Decision Letter 1

**DOI:** 10.1371/journal.pone.0329429.r003

**Authors:** Erika Kothe

29 Jun 2026

Dear Dr. Beyene,

plosone@plos.org. When you're ready to submit your revision, log on to https://www.editorialmanager.com/pone/ and select the 'Submissions Needing Revision' folder to locate your manuscript file.

- A letter that responds to each point raised by the academic editor and reviewer(s). You should upload this letter as a separate file labeled 'Response to Reviewers'.
- A marked-up copy of your manuscript that highlights changes made to the original version. You should upload this as a separate file labeled 'Revised Manuscript with Track Changes'.
- An unmarked version of your revised paper without tracked changes. You should upload this as a separate file labeled 'Manuscript'.

If you would like to make changes to your financial disclosure, please include your updated statement in your cover letter. Guidelines for resubmitting your figure files are available below the reviewer comments at the end of this letter.

If applicable, we recommend that you deposit your laboratory protocols in protocols.io to enhance the reproducibility of your results. Protocols.io assigns your protocol its own identifier (DOI) so that it can be cited independently in the future. For instructions see: https://journals.plos.org/plosone/s/submission-guidelines#loc-laboratory-protocols. Additionally, PLOS ONE offers an option for publishing peer-reviewed Lab Protocol articles, which describe protocols hosted on protocols.io. Read more information on sharing protocols at https://plos.org/protocols?utm_medium=editorial-email&utm_source=authorletters&utm_campaign=protocols.

As the corresponding author, your ORCID iD is verified in the submission system and will appear in the published article. PLOS supports the use of ORCID, and we encourage all coauthors to register for an ORCID iD and use it as well. Please encourage your coauthors to verify their ORCID iD within the submission system before final acceptance, as unverified ORCID iDs will not appear in the published article. *Only*  the individual author can complete the verification step; PLOS staff *cannot*  verify ORCID iDs on behalf of authors.

We look forward to receiving your revised manuscript.

Kind regards,

Erika Kothe

Academic Editor

PLOS One

Journal Requirements:

If the reviewer comments include a recommendation to cite specific previously published works, please review and evaluate these publications to determine whether they are relevant and should be cited. There is no requirement to cite these works unless the editor has indicated otherwise.

Please review your reference list to ensure that it is complete and correct. If you have cited papers that have been retracted, please include the rationale for doing so in the manuscript text, or remove these references and replace them with relevant current references. Any changes to the reference list should be mentioned in the rebuttal letter that accompanies your revised manuscript. If you need to cite a retracted article, indicate the article’s retracted status in the References list and also include a citation and full reference for the retraction notice.

Additional Editor Comments (if provided):

The review indicates a small change to be considered.

[Note: HTML markup is below. Please do not edit.]

Reviewers' comments:

Reviewer's Responses to Questions

**Comments to the Author**

Reviewer #3: All comments have been addressed

**********

2. Is the manuscript technically sound, and do the data support the conclusions?

Reviewer #3: Yes

**********

3. Has the statistical analysis been performed appropriately and rigorously?

Reviewer #3: N/A

**********

4. Have the authors made all data underlying the findings in their manuscript fully available?

The PLOS Data policy

Reviewer #3: Yes

**********

5. Is the manuscript presented in an intelligible fashion and written in standard English?

Reviewer #3: Yes

**********

Reviewer #3: Authors have addressed most of the comments satisfactorily. However, line no. 112-116 should be written in a better way to meet the standard of this journal.

**********

what does this mean?). If published, this will include your full peer review and any attached files.

If you choose “no”, your identity will remain anonymous but your review may still be made public.

**Do you want your identity to be public for this peer review?**  For information about this choice, including consent withdrawal, please see our Privacy Policy

Reviewer #3: **Yes:**  Dr. Kartik Chandra Guchhait

**********

[NOTE: If reviewer comments were submitted as an attachment file, they will be attached to this email and accessible via the submission site. Please log into your account, locate the manuscript record, and check for the action link "View Attachments". If this link does not appear, there are no attachment files.]

To ensure your figures meet our technical requirements, please review our figure guidelines: https://journals.plos.org/plosone/s/figures

You may also use PLOS’s free figure tool, NAAS, to help you prepare publication quality figures: https://journals.plos.org/plosone/s/figures#loc-tools-for-figure-preparation.

NAAS will assess whether your figures meet our technical requirements by comparing each figure against our figure specifications.

# Author comment: Author response to Decision Letter 2

**DOI:** 10.1371/journal.pone.0329429.r004

13 Jul 2026

10.07.2026

Point by point response to the reviewers

Arbuscular mycorrhizal fungi spore density and species diversity varied within plants species and between dry Afromontane forests in northwestern Ethiopia

PONE-D-25-33914R1

Dear Dr. Erika Kothe

Academic Editor, Plosone, and Reviewers

Many thanks for your contribution in our career development and giving this golden opportunity to revise and improve our manuscript. We highly acknowledge and appreciate your critical reviews, inputs and time invested to improve the quality of our manuscript. We learned a lot from your review comments and suggestions. We accepted all your general and specific comments and suggestion with full recognition. We have also made revision to the manuscript as per your comments and suggestions. Here below, we forward the point by point response for reviewers’ comments and suggestions.

With due respect

Belay Berza Beyene (the submitting author)

July 10.2026

Point by point response to the reviewers

Reviewer #1

Comment 1; Reviewer #3: Authors have addressed most of the comments satisfactorily. However, line no. 112-116 should be written in a better way to meet the standard of this journal.

Response to reviewer: We highly appreciate your comments and suggestions. All your comments and suggestions are incorporated accordingly in the materials and method section.( lines 112-116)

Attachment Submitted filename: Response to Reviewers.docx

# Editor report: Decision Letter 2

**DOI:** 10.1371/journal.pone.0329429.r005

**Authors:** Erika Kothe

14 Jul 2026

Arbuscular mycorrhizal fungi spore density and species diversity varied within plants species and between dry Afromontane forests in northwestern Ethiopia

PONE-D-25-33914R2

Dear Dr. Beyene,

We’re pleased to inform you that your manuscript has been judged scientifically suitable for publication and will be formally accepted for publication once it meets all outstanding technical requirements.

Within one week, you’ll receive an e-mail detailing the required amendments. When these have been addressed, you’ll receive a formal acceptance letter and your manuscript will be scheduled for publication.

An invoice will be generated when your article is formally accepted. Please note, if your institution has a publishing partnership with PLOS and your article meets the relevant criteria, all or part of your publication costs will be covered. Please make sure your user information is up-to-date by logging into Editorial Manager at Editorial Manager® and clicking the ‘Update My Information' link at the top of the page. For questions related to billing, please contact billing support.

If your institution or institutions have a press office, please notify them about your upcoming paper to help maximize its impact. If they’ll be preparing press materials, please inform our press team as soon as possible -- no later than 48 hours after receiving the formal acceptance. Your manuscript will remain under strict press embargo until 2 pm Eastern Time on the date of publication. For more information, please contact onepress@plos.org.

Kind regards,

Erika Kothe

Academic Editor

PLOS One

Additional Editor Comments (optional):

Reviewers' comments:

# Editor report: Acceptance letter

**DOI:** 10.1371/journal.pone.0329429.r006

**Authors:** Erika Kothe

PONE-D-25-33914R2

PLOS One

Dear Dr. Beyene,

I'm pleased to inform you that your manuscript has been deemed suitable for publication in PLOS One. Congratulations! Your manuscript is now being handed over to our production team.

At this stage, our production department will prepare your paper for publication. This includes ensuring the following:

* All references, tables, and figures are properly cited

* All relevant supporting information is included in the manuscript submission,

* There are no issues that prevent the paper from being properly typeset

You will receive further instructions from the production team, including instructions on how to review your proof when it is ready. Please keep in mind that we are working through a large volume of accepted articles, so please give us a few days to review your paper and let you know the next and final steps.

Lastly, if your institution or institutions have a press office, please let them know about your upcoming paper now to help maximize its impact. If they'll be preparing press materials, please inform our press team within the next 48 hours. Your manuscript will remain under strict press embargo until 2 pm Eastern Time on the date of publication. For more information, please contact onepress@plos.org.

You will receive an invoice from PLOS for your publication fee after your manuscript has reached the completed accept phase. If you receive an email requesting payment before acceptance or for any other service, this may be a phishing scheme. Learn how to identify phishing emails and protect your accounts at https://explore.plos.org/phishing.

If we can help with anything else, please email us at customercare@plos.org.

Thank you for submitting your work to PLOS One and supporting open access.

Kind regards,

PLOS One Editorial Office Staff

on behalf of

Prof. Dr. Erika Kothe

Academic Editor

PLOS One
